# The risk factors of exposure to rubella among pregnant women in Zaria 2013

**DOI:** 10.11604/pamj.supp.2019.32.1.13335

**Published:** 2019-01-21

**Authors:** Aishatu Bintu Gubio, Aisha Indo Mamman, Muhammad Abdul, Adebola Tolulope Olayinka

**Affiliations:** 1 Nigeria Field Epidemiology and Laboratory Training Program; 2 Ahmadu Bello University, Zaria, Kaduna State, Nigeria

**Keywords:** Rubella, IgG serology, vaccine

## Abstract

**Introduction:**

rubella virus usually causes a mild disease, but maternal infection early in pregnancy often leads to birth defects known as congenital rubella syndrome (CRS). Rubella remains poorly controlled in Africa despite being a vaccine preventable disease. The objective of this study was to determine the risk factor of expose of rubella and prevalence of rubella IgG antibodies among pregnant women in Zaria. The results of this study will provide data which may be used to advise the government of Kaduna State on the need to include rubella vaccine in the free routine immunization particularly for women of childbearing age.

**Methods:**

a cross-sectional study was carried out. Pregnant women attending antenatal clinics from three different health facilities in Zaria. A questionnaire was administered, to determine the proportion of pregnant women vaccinated and the sera of these women were tested for rubella IgG antibody using commercially produced enzyme-linked immunosorbent assay (ELISA) Kit. Statistical variables were compared with univariate (frequencies) bivariate (chi- square), multivariate analyses (logistic regression). A p-value of < 0.05 was considered significantly associated at 95% confidence intervals.

**Results:**

of the 246 pregnant women screened, 222 (90.2%) were positive for rubella IgG. Prevalence was highest 82/222 (36.9%) among age group 20-24 years. Those positive of those who had completed secondary school education were 104/222 (46.8%) A large number among those who tested positive with 197/222 (88.7%) were married. The Hausa tribe 155/222 (69.9%) had the highest positivity for rubella IgG. Only 2 (0.9%) women claimed to have received rubella vaccine and 159/222 (71.6%) women were seropositive for IgG among the unemployed group.

**Conclusion:**

the serological evidence of rubella virus is an indication that rubella is endemic in Nigeria. Nigeria should include rubella vaccination in the routine immunization exercise for women before they get pregnant to reduce the risk of CRS.

## Introduction

During the first trimester of pregnancy, rubella virus may infect all organs of the fetus which could cause severe birth defects, death or abortion described as Congenital Rubella Syndrome (CRS) [1–3]. Rubella virus being the only member *Rubivirus* and the family *Togaviridae* is commonly known as three-day measles or German measles [2] and symptoms of rubella include low-grade fever, malaise, lymphadenopathy, upper respiratory symptoms, sore throat, maculopapular rash [3]. The disease is transmitted through respiratory droplets or transplacentally. The incubation period is 14 days [3]. The prevalence of rubella immunity varies in different geographical areas of the world. Although women have a relatively higher prevalence of rubella immunity (93.2%) in Europe [4], cases of multiple rubella defects recognizable close to the time of birth in infants in the United Kingdom have been reported [5].

In Africa, there is a general lack of awareness of rubella and its prevalence therefore not often diagnosed. A community-based cluster sample survey of rubella seroepidemiology in Addis Ababa, Ethiopia showed a rubella antibody prevalence of 91% among 466 individuals [6]. Rubella is currently not included in the routine immunization program in Nigeria [7]. In Lagos State Nigeria, a study on rubella-IgG antibody in women of childbearing age gave a seropositivity rate of 77% [8]. While in Maiduguri, a study among pregnant women showed that 46% of the women had evidence of acute infection and were IgM positive [9]. IgG rubella antibodies were found in 421 of 430 (97.9%) pregnant women in Ahmadu Bello University Teaching Hospital Zaria between May 2007 and February 2008 [2]. In Oyo State Nigeria 215 (93.5%) of the women screened for rubella IgG turned out positive [10]. Studies conducted in Oyo State by Adesina *et al*. (2008) have shown that over the years more women are becoming exposed to rubella virus. Rubella poses a great economic burden on countries affected due to increased cost of managing children with CRS hence the need to protect the unexposed population [10]. Rubella is a vaccine preventable disease, and vaccination is the best preventable measure for protection against rubella and CRS outbreaks [10,11] The United States reported rubella elimination in the country in 2004 [3]. Live, attenuated rubella vaccines have failed to be adopted in developing countries for over 30 years and rates of CRS remaining unchanged in developing countries of the world [11]. We set out to determine the seroprevalence and risk factors of rubella virus antibodies IgG and IgM among pregnant women in Zaria, Northern, Nigeria.

## Methods

**Study area:** the study was conducted between February 2012 and November 2013 in Zaria metropolis which is about 300km from the Abuja Capital Zaria metropolis has two Local Government Areas (LGA) - Sabon Gari and Zaria with a total population of about 2 million people their main economic activity is agriculture. The study was conducted in three health facilities which are: one Tertiary Hospital (TH) Ahmadu Bello University Teaching Hospital Zaria, one General Hospital (GH) Major Abdullahi Memorial Hospital, located at Sabon Gari; and one Primary Health Care (PHC) Tudun Wada in Zaria LGA.

**Study design:** the study design was cross-sectional. A total of 246 blood samples were collected by random number generation from women who were pregnant that gave their consent to participate as they came in to attend antenatal clinics at the above health care facilities in Zaria metropolis.

**Sample size determination:** the minimum sample size was obtained using the formula for cross-sectional study designs [9] and a prevalence of 80% [12] which provided a minimum sample size of 246.

**Study population:** pregnant women aged 15 years to 49 years who were attending antenatal clinics at the selected health care facilities in Kaduna State.

**Inclusion criteria:** apparently healthy pregnant women between 15years to 49years attending ante- natal clinic at the selected health care facilities.

**Exclusion criteria:** women who were pregnant but did not want to participate and pregnant women not attending ante-natal care at selected health care facilities Women who are < 15 or > 49 years.

**Sampling technique:** the health facilities in Nigeria have been categorized into Primary, Secondary and Tertiary Health Care Facilities. Ahmadu Bello University Teaching Hospital (ABUTH) Zaria been the only tertiary hospital was selected automatically. There are two secondary facilities in Zaria one was selected based on purposive sampling technique which was the number of highest volume of patients attending the health facility compared to the other health facility and out of the fifteen Primary Health Care facility available in Zaria one was also selected base on purposive sampling technique which was the number of highest volume of patients attending the facility compared to the other health facility.

**Study instrument:** questionnaires which was written in English and translated to Hausa were administered to the pregnant women where applicable to obtain demographic information and possible risk factors associated with rubella virus antibodies IgG and IgM. Blood samples were also collected from consenting participants.

**Data collection methods:** about 5 ml of blood was collected from each pregnant woman using a sterile syringe and transferred into plain sample bottles. Sera was extracted and stored at -20ºC at the Hematology Laboratory of ABUTH Zaria.

**Procedure for serum analysis:** antibody tests against rubella IgG were carried out by Enzyme-Linked Immunosorbent Assay (ELISA) (ELISA kit: Axiom diagnostics Siegfridstr, 14, 67,547 Worms Germany). Samples with absorbance values greater or equal to the absorbance of the standard were considered positive while absorbance less than absorbance of the standard are considered negative. The absorbance was read at 490nm using microtitre plate and the cut-off absorbance standard was set at 0.18 for rubella.

**Statistical analysis:** we collected data using Microsoft Excel and exported to Epi InfoTM version 3.5.3 (Centers for Disease Control and Prevention, Atlanta USA) was used to analyze results [13]. Independent variables are the age, sex, educational status, employment status. The dependent variables are antibody IgG, IgM positive or negative. Categorical variables were compared with univariate, bivariate, multivariate analyses. A p-value of < 0.05 was considered significant at 95% confidence intervals.

**Ethical issues:** ethical approval was obtained from the Ethical Committee of the Ahmadu Bello University Teaching Hospital Zaria. Permission was obtained from the Local Government Area Council Zaria. While verbal and written consent was obtained from all participants. Children below the age of consent were consented with by their guardians while the children assented.

## Results

### Socio-demographic of pregnant women attending antenatal clinic in Zaria 2013

Of the 246 pregnant women enrolled, a total of 112 (45.5%) of the women had secondary education and 180 (73.2%) of them were housewives. The majority of the women were Muslims 211 (85.8%). Only two of them (0.8%) had ever received rubella vaccine (Table 1).

**Table 1:** socio-demographic of pregnant women attending Antenatal clinic in Zaria 2013

Characteristics	Frequency (n = 356)	Percent (%)
**Age group (years)**		
15 – 19	33	13.4
20 – 24	90	36.6
25 – 29	66	26.8
30 – 34	31	12.6
35 – 39	21	8.5
40 - 49	5	2.0
**Educational status**		
Primary	63	25.6
Secondary	112	45.5
Tertiary	41	16.7
None	30	12.2
**Employment status**		
Employed	8	3.3
Unemployed	180	73.2
Self-employed	58	23.6
Religion		
Islam	211	85.8
Christian	35	14.2
**Tribe**		
Hausa	174	70.7
Fulani	19	7.7
Yoruba	21	8.5
Others	32	13.0
**Marital status**		
Married	215	87.4
Divorced	13	5.3
Single	11	4.5
Widowed		2.8
Previous Vaccination	7	
Don’t know	70	28.5
No	174	70.3
Yes	2	0.8

### Laboratory results of pregnant women attending ante-natal clinic in Zaria 2013

A total of 222/246 (90.2%) of the women were rubella IgG positive. Women between the ages 20-24 years had the highest rubella IgG positivity with 82/222 (36.9%) and lowest among the age group 40-47 4/222 (1.8%). Those with secondary education had the highest rubella IgG seropositivity 104/222 (46.8%) while those who didn’t go to school had the lowest 27/222 (12.2%). Rubella IgG positivity was highest in those who were married with 197/222 (88.7%) positivity rate (Table 2).

**Table 2:** laboratory results of pregnant women attending ante-natal clinic in Zaria 2013

	IgG Negative		IgG Positive		
Characteristics	No.	%(%)	No.	%	Chi Square
**Age group(years)**					
15 - 19	3	9.0	30	91.0	-
20 - 24	8	9.0	82	91.0	0.00
25 - 29	6	9.0	60	91.0	0.00
30 - 34	5	16.0	26	84.0	0.72
35 - 39	1	5.0	20	95.0	0.35
40 - 49		2.0	4	80.0	0.55
**Educational status**	1				
Primary	11	17.0	52	83.0	-
Secondary	8	7.0	104	93.0	4.44
Tertiary	2	5.0	39	95.0	4.59
None		10.0	27	90.0	0.88
**Employment status**	33				
Employed	0	0.0	8	100.0	-
Unemployed	21	12.0	159	88.0	1.05
Self-employed	3	5.0	55	95.0	0.43
**Religion**					
Islam	21	10.0	190	90.1	-
Christian	3	9.0	32	91.0	0.07
**Tribe**					
Hausa	19	11.0	155	89.0	-
Fulani	3	16.0	16	84.	0.40
Yoruba	0	0.0	21	100.0	2.54
Others	2	6.0	30	94.0	0.64
**Marital status**					
Married	18	8.0	197	92.0	-
Divorced	1	8.0	12	92.0	0.01
Single	4	36.0	7	64.0	9.33
Widowed		14.0	6	82.0	0.30
**Previous Vaccination**	1				
Don’t know	13	19.0	57	81.0	-
No	11	6.0	163	94.0	8.45
Yes	0	0.0	2	100.0	0.45

### Factors associated with rubella IgG antibody among pregnant women

The highest positivity for rubella IgG 159 (71.6%) occurred among the unemployed group. However, there was no statistically significant association between rubella seropositivity and employment status [OR 2.77; 95% CI (0.80-9.63)]. The rubella seropositivity rate was the same among educated and non-educated women at 195 (90.3%) of those educated and 27 (90.0%) of those uneducated [OR1.03; 95% CI (0.29-3.68)]. (97.6%) pregnant women had a history of stillbirth, and were positive for rubella IgG, p-value = 0.08) (Table 3).

**Table 3:** factors associated with rubella IgG antibody among pregnant women

Characteristics	IgG Negative		IgG Positive		POR (95% CI)^+^ No.	P-Value
	No.	%	No.	%
SocioeconomicStatus						
Employed	3	4.5	63	95.5	2.77(0.80-9.63)	0.10
Unemployed	21	11.7	159	88.3	1.03(0.29-3.68)	
Educational Status						
Educated	21	9.7	195	90.3		0.96
None	3	10.0	27	90.0		
No. of Deliveries						
0-7	16	10.8	132	89.2	0.73(0.30-1.79)	0.49
8-14	8	8.2	90	91.8		
History of Still birth						
Yes	1	2.4	40	97.6	5.05(0.66-38.54)	0.08
No	23	11.2	182	88.9		
History of Vaccination						
Yes	24	9.8	220	90.2	0.218(0.64-0.46)	0.01
No	0	0.0	2	0.9		
Total	24	100	222	90.2		

+ POR Prevalence Odds Ratio

## Discussion

Our study showed a high prevalence of rubella IgG antibodies among pregnant women attending antenatal clinics in Zaria. This finding is similar to the finding of Muhammad *et al*. (2010) whose study showed a 97%, Bamboye *et al*. (2004) 95.5%, Olajideet *et al*. (2012) 93.1%, IgG rubella seropositivity. The high prevalence could be as a result of previous exposure to the rubella virus and lack of vaccination due to rubella [12]. The vaccination rate against rubella virus was very low. Rubella is currently not included in the routine immunization program in Nigeria [7]. The primary purpose of rubella vaccination is the prevention of congenital rubella infection including CRS. In 2013 WHO reported that the incidence of rubella virus infection and CRS had reduced drastically in developed countries due to the introduction of rubella vaccination in their national routine immunization program [14].

The highest age-related seropositivity rate for rubella IgG was between ages 20-24. Other studies suggested that women of this age group are sexually active and this may probably predispose them to greater risk of exposure and infection [12]. The presence of the IgG antibodies indicates a past infection and the antibody levels is linked to B-memory cell activity [15]. The lowest seropositivity rate fell between ages 40-47 years for rubella IgG this explains that most women between this age group have been exposed to rubella virus. This secondary education group accounting for 104 (46.8%) had the highest seropositivity rate for IgG. This finding which was similar to 54.1% obtained by the Maiduguri secondary education group [8] could have been attributed to lack of awareness of rubella virus among this group. Rubella virus continues to circulate elsewhere in the world, especially in regions where rubella vaccination programs have not been established (e.g. the African Region) [15]. In Nigeria, only a few surveys have so far been conducted [8]. This study like other previous studies has shown that a high proportion of our population has rubella immunity suggesting exposures to previous immune attacks. Babalola (2004) reported that all immune Nigerians acquire their immunity by natural infection [16]. This study reinforces the few existing information on the distribution of rubella immunity in Nigeria. Even though some respondents had recall bias, the questionnaire was able to frame questions by asking if their parents took them to the hospitals when they were young or were allowed to receive injection to capture this limitation.

## Conclusion

Seroprevalence of rubella virus antibodies was high among pregnant women in Zaria. We, therefore, recommend to the Kaduna state Ministry of Health that pre-pubertal girls should be screened and vaccinated against rubella before getting married or pregnant, and pregnant women should be educated on rubella virus during ANC. The Federal State Ministry of Health, stakeholders and community leaders should create awareness on rubella virus infection.

### What is known about this topic

- The prevalence of rubella immunity varies in different geographical areas of the world;
- During the first trimester of pregnancy, rubella virus may infect all organs of the fetus which could cause severe birth defects, death or abortion described as Congentital Rubella Syndrome (CRS);
- Rubella is a vaccine preventable disease and vaccination is the best measure for protection against rubella.

### What this study adds

- The sero prevalence of rubella antibodies among pregnant women in Zaria was 90.2%;
- Less than 1% of the women surveyed said they had received rubella vaccine and 70% could not tell whether they had received it or not.

## Competing interests

The authors declare no competing interests.
